# The Role of Autonomy in Defining Work Arrangements and Its Effect on Psychological Safety

**DOI:** 10.21500/20112084.7492

**Published:** 2025-11-10

**Authors:** Milena da Silva-Guimaraes, Pedro Fialho, Carolina Moliner, Monique Delfim-Andrade

**Affiliations:** 1 Universitat de Valencia. Valencia, España. Universitat de Valencia Universitat de Valencia Valencia Spain; 2 Universidade de Coimbra. Coimbra, Portugal. Universidade de Coimbra Universidade de Coimbra Coimbra Portugal

**Keywords:** Psychological Safety, working arrangement, teleworking, hybrid work, in-person work, Seguridad psicológica, arreglo laboral, teletrabajo, trabajo híbrido, trabajo presencial

## Abstract

This study examines the interaction between work arrangements (in- person, remote, or hybrid) and autonomy in defining this arrangement on perceived psychological safety in Brazilian workers (N = 506; 300 women, 206 men). This study adopted a cross-sectional design, data were collected in a non-probabilistic convenience sampling, the measures used were psychological safety, working arrangements and autonomy. The analyses included independent samples t-tests and analysis of variance (ANOVA). The findings indicated that psychological safety increased with virtuality levels. Interestingly, when companies choose to implement remote and hybrid work arrangements, employees felt more psychologically safe than when they chose to do so voluntarily. This research suggests that organizations committed to remote work arrangements promote the perceived psychological safety of their workers. Employees may feel that, by establishing a telework arrangement, the organization demonstrates trustworthy behavior toward them.

## Resumen

Este estudio examina la interacción entre las modalidades de trabajo (presencial, remoto o híbrido) y la autonomía en la definición del acuerdo laboral sobre la seguridad psicológica percibida en trabajadores brasileños (N = 506; 300 mujeres, 206 hombres). Este estudio adoptó un diseño de investigación transversal. Los datos se recolectaron mediante un muestreo no probabilístico por conveniencia. Las variables medidas fueron seguridad psicológica, modalidades de trabajo y autonomía. Los análisis estadísticos realizados incluyeron pruebas t y análisis de la varianza (ANOVA). Los resultados mostraron que a mayor nivel de virtualidad, mayor fue el nivel de seguridad psicológica. Sorprendentemente, en el caso de los trabajadores remotos e híbridos, la seguridad psicológica fue mayor cuando las organizaciones decidieron adoptar estas modalidades de trabajo que cuando los empleados decidieron hacerlo voluntariamente. Esta investigación sugiere que las organizaciones que se comprometen con acuerdos de trabajo remoto parecen promover la seguridad psicológica percibida de sus trabajadores. Los empleados pueden sentir que, al definir un acuerdo de teletrabajo, la organización demuestra un comportamiento de confianza hacia ellos.

## 1. Introduction

Working arrangements have been changing constantly. In this scenario, the terms of employment should reflect a mutual understanding between the employer and the employee, with mutual agreement on the work location, schedule, deadlines, reporting methods, and other relevant details (ILO, 2020; Kossek et al., 2015; Peters et al., 2016).

In today's complex work environment, it is common for employees to be proactive in their roles, contribute to their teams, develop themselves, and participate in various other behaviors that promote psychological safety, such as taking risks, learning from mistakes, and accepting feedback from others (Edmondson, 2019). In this evolving landscape, psychological safety - a perception related to the consequences of taking interpersonal risks, most notably at work (Edmondson, 1999) - becomes crucial for employee development and workplace comfort (Edmondson & Bransby, 2023; Edmondson & Lei, 2014). Recent research on psychological safety in telework settings (Edmondson & Bransby, 2023; O'Donovan & McAuliffe, 2020) highlights the need to understand how different working arrangements affect employee well-being and organizational effectiveness. Following this research line, this study exami nes how employee autonomy in defining work arrangements interacts with psychological safety across different work arrangements (in-person, remote, and hybrid). By investigating the relationship between autonomy in defining work arrangements and psychological safety across in-person, remote, and hybrid settings, this research provides insights to inform evidence-based policies for managing contemporary work environments.

### 1.1 Literature Review and Hypothesis Formulation

The literature indicates that most of employees currently choose flexibility rather than economic and material gains (Reisinger & Fetterer, 2021).

Previous studies (e.g., Deci & Ryan, 1985a; Edmondson, 1999; Rousseau et al. 1998; Ryan & Deci, 2017) show that people may perceive that the more autonomy they have, the more voluntarily they can choose and the more trusted they feel. In this sense, when leaders grant employees autonomy over their work arrangements, they can be showing a positive expectation about their work arrangements, they can be showing a positive expectation about their employees' intentions and capabilities, thereby fostering the trust relationship.

This can be also related to Self Determination Theory (Deci & Ryan, 1985; Kibaroglu et al., 2025), which focuses on how social contexts and interpersonal interactions either facilitate or undermine intrinsic motivation (Ryan & Deci, 2000). Intrinsic motivation means engaging in activities for their inherent enjoyment and satisfaction, influenced by the degree of autonomy and competence perceived by individuals (Ryan, 2009). When social environments support these needs, intrinsic motivation is enhanced (Newman et al., 2017; Ryan & Deci, 2000). In this sense, we believe that allowing employees to have autonomy to de fine their working arrangements may play a critical role that may bolster an employee's intrinsic motivation and promote an environment with psychological safety.

As mentioned before, psychological safety is a perception related to the consequences of taking interpersonal risks, most notably at work (Edmondson, 1999). In such an environment, individuals feel secure in taking interpersonal risks, such as seeking feedback, sharing information, and experimenting with new ideas (Edmondson, 1999). (Edmondson, 1999).

Supporting this theoretical framework, re cent empirical evidence demonstrates the practical implications of these concepts in modern work environments. Psychological safety can also be developed in virtual teams, in this sense, when there is a commitment by the organizations, these environments are able to promote comfort and a safe space for employees so they will feel more prepared to also take interpersonal risks in virtual teams (Dzandu et al., 2023). Based on these findings, we can interpret that when organizations provide flexible working arrangements, employees may develop greater trust in their workplace relationships, which subsequently translates into higher levels of psychological safety. This reinforces the connection between organizational autonomy support and the creation of psychologically safe work environments.

Therefore, we consider that employees may think that when a company gives them the autonomy to choose their working arrangement, the company may trust them, which can be extend to other areas, such as the developing of sincere and positive relationships with other team members, and providing feedback, actions that can promote psychological safety.

Furthermore, the psychological dynamics of work arrangement decisions may be influenced by social comparison processes. Przybylski et al. (2013) mention the "fear of missing out" (FoMO), understood as a concern that, while one is absent from a space or experience, others are probably having more opportunities, ad- vantages, or benefits that one is missing. This concept may be particularly relevant in the context of flexible work arrangements, where employees' sense of inclusion and connection to their workplace can be affected by their physical presence or absence. Given this, we propose the following hypothesis:

H1: Perceived psychological safety is higher when workers have autonomy in defining work arrangement.

Recent studies (e.g., Lechner & Mortlock, 2021; Smith, 2022) show that remote and hybrid em- ployees may experience higher psychological safety levels than employees who operate in traditional working paradigms. It is important to mention that, although telework brings new challenges to promote psychological safety in virtual teams, it also brings possibilities to develop it, for example, as individuals are physically distant, some people may feel more comfortable to speak or ask questions to a colleague or the leader. The same ha- ppens when people report that feel more comfortable online rather than face-to-face interactions. Thus, the following hypothesis is formulated: 

H2: Perceived psychological safety is higher in hybrid and/or remote working arrangements, comparing to in-person working arrangements.

The world of work is constantly changing and this drives organizations to change their operational practices to adjust to the increasing integration of technology and evolving organizational structures. The implementation of remote work arrangements and employees' autonomy can be examples of this adjustments, which can bring challenges but also benefits. Some beneficial outcomes for both organizations and employees were identified within the relationship between teleworking and autonomy, to name a few, customer satisfaction, employee job satisfaction, workplace engagement, and organizational commitment (Theurer et al., 2018).

In the working environment, autonomy can refer to the employees' subjective experience of freedom, temporal independence, task completion, and decision-making capacity regarding their work responsibilities (Stegmann et al., 2010). In this study, we understand autonomy as a necessary advancement, boosted by the Covid-19 pandemic, that allows employees to have more flexibility at work and can positively impact in their wellbeing. However, it becomes imperative to examine whether employees have genuinely attained autonomy through their selection of preferred work arrangements or if they are being pressured by their leaders and peers (Tanzi, 2022).

In light of these considerations, we aim to examine whether perceived psychological safety varies in association with autonomy across three distinct work arrangements: in-person, hybrid, and remote. Accordingly, the following hypothesis is proposed:

H3: Perceived psychological safety is higher in groups when employees have autonomy in difining hybrid and remote working arragements.

## 2. Method

### 2.1 Participants

The study included 506 participants who provided informed consent and were guaranteed anonymity. This study adopted a cross-sectional design, and non-probabilistic convenience sampling, that comprised primarily Brazilian employees (M = 34.3, SD = 9.4), with 59.2% female and 40.8% male participants, most holding postgraduate (54%) or bachelor's degrees (39.5%). Most worked under permanent con- tracts (86.6%), organizational tenure of 5.44 years (SD = 6.2). Regarding working arrangements, 53.8% worked in hybrid settings, 28.7% remotely, and 17.5% in-person, with 90.3% of arrangements decided by organizations rather than employees. Most participants (77.1%) considered their working arrangement adequate, with hybrid (51.4%) and remote (43.8%) being the preferred arrangements. Close to one-third (26.6%) held management positions.

Inclusion criteria required participants to be currently employed in Brazil and working under one of the three work arrangements studied.

### 2.2 Measures

#### 2.2.1 Working Arrangement

Working arrangements were assessed by asking participants to select their current work model: in-person, remote (work performed via information technologies), or hybrid (combination of in-person and remote). Participants were categorized into two groups based on who determined the arrangement: organization-decided or employee-decided.

#### 2.2.2 Autonomy

Autonomy was measured by asking participants who decided their working arrangement: organization/employer or employee's decision.

#### 2.2.3 Psychological Safety

We measured psychological safety through the Edmondson (1999) 7-item scale of Team Psychological Safety, whose Portuguese version was used by Gari et al. (2020). A sample item is: “It is safe to take a risk on this team.” The items were presented on a Likert scale, varying from 1 (very inaccurate) to 7 (very accurate). McDonald{s for this measure was .72.

### 2.3 Data Analysis

T-test, one-way analysis of variance (ANO VA), and two-way ANOVA were used to analyze the data using the software SPSS 28 and Jamovi 2.3.21.

#### 2.3.1 Ethical Considerations and Procedure

An online survey in the Portuguese language was used to gather data. The tool was distributed mostly via social media, particularly LinkedIn, and was co-owned by a research team looking into telework. Employees of major Brazilian corporations were invited to participate in the study, and an acti ve search for possible responders was also carried out. Every invitation included information on the study's goals, requirements for participation, time-line for completion, and confidentiality guarantees.

After giving their consent for the use and confidentiality of their data, participants completed two screening questions that determined whether they were now employed in Brazil and working under one of the three work arrangements studied. The qualified respondents filled out the sections on sociodemographic, work arrangements, and psychological safety.

## 3. Results

Levene's Test of Homogeneity of Variance was applied to the groups under comparison: autonomy (decisions made by the company and employees) and working arrangements (in-person, hybrid, and remote). The data meet the homogeneity of variance assumption (F=.53, p=.75). Homoscedasticity was confirmed, and the variance of all the groups under comparison is the same.

### 3.1 Confirmatory Factorial Analysis (CFA) of Psychological Safety Scale

Running a CFA showed that all the items from the psychological safety scale were considered statistically significant (p<.001), and SRMR= .06. However, x^2^/df = 7.64, CFI and TLI equal to .84 and .76 respectively, and RMSEA= .12. Modification indices suggested correlating error terms for items 5 and 3 (MI = 29.84), and items 5 and 1 (MI = 26.32).

They correspond to the three inverted items in the psychological safety scale. We adjusted the model with the modified items, and better results were presented: CFI=.93, TLI=.89, SRMR=.05, RMSEA=.08, and x2/df=4.23., although they were not as satisfactory as expected. Ex- cellent fit was achieved when the three in verted items were separated from the re gular items, as follows: CFI=.98, TLI=.95, SRMR=.02, RMSEA=.07, and x2/df=3.50.

### 3.2 Validity and Reliability

The initial CFA model showed poor fit (X2 = 85.5, df= 14, p > .001 ; RMSEA = .116; CFI = .845; TLI = .767; SRMR = .066). After correlating residuals between items 1, 3, and 5 (reversed items), the model achieved excellent fit (X2 = 21.5, df = 11, p = .028; RMSEA = .050; CFI = .977; TLI = .956; SRMR = .032). The scale demonstrated acceptable reliability (w = .72).

### 3.3 Hypotheses Testing Analyses

To test the first hypothesis that perceived psychological safety is higher when workers have autonomy in defining work arrangement, we conducted a t-test of independent samples to prove the existence of differences between the two groups. Differences between the groups were to be anticipated. The findings, however, indicated that the employee's decision and the organization's decision did not differ statistically significantly (t(504)= -1.09, p>.05). Perceived psychological safety was somewhat greater in the employee's decision group (M=5.4317, SD=.96866) than in the organization group (M=5.2656, SD=1.00546).

However, the first hypothesis was not supported and according to this study, autonomy does not predict psychological safety.

In the second hypothesis - perceived psychological safety is higher in hybrid and/or remote working arrangements, compared to in-person working arrangements. To determine if the three groups of working arrangements (in-person, remote, and hybrid) and psychological safety differed significantly from one another, an ANOVA was performed (see Appendix - Table 1). The findings revealed notable variations among the three working arrangements and that psychological safety is higher in telework arrangements (hybrid and remo te), F(2,460)=9.18, p<.001. Thus, the second hypothesis was supported.

A post hoc test was used to determine which groups the differences were significant for (Table 1). The post hoc test indicates that there are significant differences (p< .001) across the resulting groups in-person (M=5.00, SD=1.03) and remote (M=5.56, SD=.93378), also between hybrid (M=5.22, SD=.99) and remote (M=5.56, SD=.93) working arrangements (p<.003).

**Table 1:** Differences between working arrangements and psychological safety.

Working arrangements	Mean Difference	Sig.
Remote vs In-person	.56	p<.001
Remote vs Hybrid	.34	p<.003

This table shows a performed post hoc test to clarify which working arrangements presented significant mean differences.

**Figure 1:**
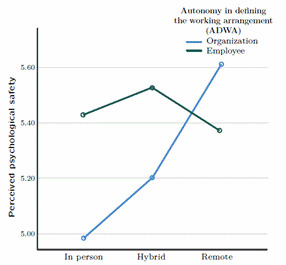
Interaction model between ADWA, work arragements and psycological safety.

 To the hypothesis three - perceived psychological safety is higher when employees have autonomy in defining hybrid and remote working arrangements - We predicted that when employees have the autonomy to choose whether to work remotely or hybrid, they will feel more psychologically safe. To test this hypothesis, we performed a factorial ANOVA for independent groups and looked at whether there were any significant differences between the variables or the combination of the va riables. We previously checked for the assumptions and we have a robust sample size. Pairwise com- parisons indicated statistically significant differences (p<.001) between remote (M=5.61, SD=.09) and in-person (M=4.98, SD=.11) working arrangements. Similarly, between hybrid (M=5.19, SD=.06) and remote (M=5.61, SD=.09) groups, p<.001. Nevertheless only when the organization chooses the working arrangement do the distinctions become important., as shown in Appendix (see Table 2 and Figure 1). Therefore, the third hypothesis was partially supported.

**Table 2:** Significant differences between working arran- gements when defined by the organization

Autonomy	Working arrangements	Mean Difference	Sig.
Organization	Remote vs In-person	.63	p<.001
	Remote vs Hybrid	.41	p<.001

*This table portrays pairwise comparisons showing the significant mean differences between working arrangements when the organization decides.

## 4. Discussion

The present study aimed to understand the role of autonomy on psychological safety among employees from different working arrangement realities. Ultimately, we wanted to understand how employees reported different levels of psychological safety, whether they worked in-person, remotely, or in a hybrid environment, and whether they chose their working arrangement or the organization did. Our first hypothesis was that perceived psychological safety was higher when workers have autonomy in defining work arrangement, however, in this study, autonomy does not predict psychological safety. The fact that nearly one-third of the sample had a managerial or leader-ship role could be one reason, suggesting that they adhere to the organization's prior working policies. Furthermore, the majority of respondents work remotely or in hybrid arrangements, which could give the impression that these arrangements already include some degree of autonomy, whether chosen by the individual or the company.

The second hypothesis - perceived psychological safety is higher in hybrid and/or remote working arrangements, comparing to in-person working arrangements - was confirmed and our findings showed significant differences in perceived psychological safety across in-person, remote, and hybrid working arrangements. The findings confirmed that there are notable distinctions between hybrid and remote groups as well as between remote and in-person working arrangements. This followed our expectations and aligned with previous studies (Dzandu et al., 2023; Smith, 2022; Tanzi, 2022), showing that currently, employees prefer and may feel more psychologically safety on remote and hybrid working arrangements.

The third hypothesis proposed that perceived psychological safety is higher when employees have autonomy in defining hybrid and remote working arrangements. The results show that perceived psychological safety increases on remote or hybrid working arrangements. If employees are more inclined to these arrangements, they may feel more comfortable in their working environment and their relationships with their team members, which explains the higher levels of psychological safety. Surprisingly, though, the distinctions in both situations only become important when the company, not the person, makes the decision to embrace teleworking. This might be a factor in determining whether or not someone believes that their workplace environment provides them with the trust they need to permit teleworking alternatives. The psychological safety that the individual feels in the team they join is likely to be influenced by that trust. Furthermore, it can also be explained by the psychological contract. According to Rous seau (1989), the psychological contract emerges when the individual expects reciprocity from the organization in response to his contributions. This expectation of reciprocity is only possible based on a pre-existing relationship between the parties. In addition, the psychological contract concept focuses on the employee's experience. Another possible explanation could be related to organizational trust. In this study, we can relate it to the issue of reciprocating trust, which can be a rebalancing of the psychological contract (Rousseau et al., 1998). If the company gives trust to the employees, they may feel the need to give something back (trust via psychological safety). By establishing a working arrangement that includes a remote component, employees might believe that the company is acting in a trustworthy manner toward them. Furthermore, the remote/hybrid model may foster psychological safety that spreads throughout the entire organization since it necessitates open and honest communication between the employee and the company in order to support work execution.

This perception can even be linked to the "fear of missing out" or "feeling of missing out" (FoMO), which is defined as the persistent fear that others may be having fulfilling experiences while one is not present and is explained by the desire to continuously stay in touch with what others are doing (Przybylski et al., 2013). When the organization defines a working arrangement with some remote component, the employee might not feel his participation is being missed because every employee works in the same arrangement. On the other hand, an employee may feel left out if he chooses to work remotely while his coworkers choose to work in person.

According to the findings, Perceived psychological safety tends to decline in the groups that the company selects for the in-person working arrangement, according to the study's findings. Employees may view this differently. For instance, they may interpret it as a lack of trust in the company because they feel that monitoring employees in person is easier than doing so online. A company's decision to solely use the in-person approach could also be seen as a lack of trust, given the growing popularity of remo te and hybrid working arrangements.

## 5. Theoretical Implications

Teleworking arrangements require specific competencies to address those environments' needs, reduce virtual distance, and create beneficial environments (e.g., trustful and safe) (Reilly & Lojeski, 2020). Edmondson and Bransby (2023) reviewed 185 papers regarding psychological safety; more than half of these studies were published between 2019 and 2021. It is interesting to notice how research on psychological safety has increased these last years, the years most impacted by the COVID-19 pandemic and, consequently, the years where teleworking was most discussed and implemented.

Since many of these changes in the workplace are new, we do not yet know how these working arrangements may affect employees or how they may affect businesses. Under the current working arrangements, we do not know if people feel psychologically safe in their workplace. To date, psychological safety has only been utilized as an outcome variable in a small number of research that have looked at it in the context of telework. Given the timing of the- se discussions, this study contributes to the literature regarding autonomy, teleworking arrangements, and psychological safety.

## 6. Practical Implications

The findings indicate that psychological safety increases with the degree of virtuality. When companies choose to implement remote work arrangements, psychological safety was higher than when employees made the decision on their own. Accor- ding to this study, remote working policies have a fa vorable impact on employees' perceptions of psycho- logical safety and an organization's ability to survive, particularly when those policies are implemented.

These findings contribute to support organizations that are considering to implement or maintain telework arrangements. This study shows that it is possible to develop psychological safety in virtual teams when the organization is truly committed to telework and to actions that help maintain a good communication and relationships with the employees.

In this sense, organizations that proactively implement teleworking arrangements may foster a stronger sense of psychological safety among their employees compared to companies where employees individually choose to work remotely. This can show the relevance of organizational commitment and structured implementation of remote work policies.

Concerns regarding the possible harmful influence of remote work on psychological safety are widespread. However, organizations can implement strategies to maintain interpersonal connections online, and even improve psychological safety. To name a few strategies: regular virtual check-ins, team-building events, and open communication can help preserve a sense of connection and support among physically distant team members (Edmondson, 2019).

Companies should develop straightforward policies and procedures encouraging remote work, such as flexible scheduling, access to essential equipment, and virtual communication protocols. These methods can help employees feel supported and comfortable in remote work settings (Kossek et al., 2015).

It is critical to train managers and staff on appropriate remote work practices and interaction strategies. This can involve training on digital technologies, remote collaboration techniques, and methods for maintaining work-life balance (Peters et al., 2016). In conclusion, this study's findings highlight the potential benefits of remo te working arrangements for fostering psychological safety. Organizations that commit to and promote remote work may establish a work environment where workers feel secure and connected. Companies may effectively manage and capitalize on the benefits of remote work by addressing issues through innovative initiatives and supporting policies.

## 7. Limitations and Future Studies

The current study provides noteworthy contributions to the literature; however, there are also several limitations. First of all, there is a reduced number of participants in the group where the employees could make a decision. Second, organizations have only recently broadly adopted remo te and hybrid working arrangements, so people may still adjust, which means that the perception of the employees will probably still change, some- times and under certain circumstances considering them in a positive or negative way.

Third, data collection was done through an online survey, with the dissemination strategy mostly using the LinkedIn. In addition, participation was voluntary, and the sample size of 506 was therefore not representative of any particular company, sector, or country. In this sense, we lack sufficient contextual knowledge about the population under the study as the results are difficult to generalize due to the sampling method. Moreover, nearly a third of our sample held a manager position, who probably have different visions regarding autonomy, the working arrangement's contents, and psychological safety in comparison to their subordinates.

We should have used more questions to evaluate autonomy and included flexibility-related aspects, which could have been more informative and pre cise. Employee preference should have also been considered in this study. In addition, it would have been interesting to ask the percentage of remote work (e.g., in a range), especially for employees who work in a hybrid work arrangement, as the work characteristics and perception can change entirely for someone who works 30% remote or 90% remo te, even though both arrangements are hybrid.

Although this work has limits, it has intriguing theoretical and practical impacts. Theoretically, the present study reinforces that remote and hybrid working arrangements may positively predict perceived psychological safety. Businesses that currently invest in these working arrangements and those who might be reluctant to do so out of fear that something might go wrong might do so in the future by putting the right programs and initiatives in place.

As previously stated, additional research in the literature is required to examine the relationship between psychological safety, the working arrangements, and autonomy. Given that our sample is predominantly composed by Brazilian employees, it could be interesting that this topic be further investigated with a bigger sample size and possibly with employees of different nationalities. Data collected from several nations may therefore produce disparate findings.

Future study along this line may also examine productivity and performance in the three types of working arrangements and how it affects psychological safety perceptions. Additionally, to deter mine whether a relationship between felt psychological safety and working arrangements would foster an innovative and creative work environment.

Carrying out the quantitative study with more participants and increasing the sample size is a second via ble investigation, given that some of the findings in this study might be limited by the fact that the sample was partially reduced. In a time when working remotely and in a hybrid environment is more popular, research is also being done that aims to connect psychological safety to factors like organizational trust.

## 8. Conclusions

This study aimed to understand how employee autonomy in defining the work arrangements interacts with psychological safety across different work arrangements (in-person, remote, and hybrid). By investigating the relations- hip between autonomy in defining work arrangements and psychological safety across in-person, remote, and hybrid settings, this research intended to provide insights to inform evidence-based policies for managing contemporary work environments.

The results highlight that, in this study, autonomy in defining the work arrangements does not directly predict psychological safety. However, our findings revealed significant differences in psychological safety levels across work arrangements, with higher levels observed in remote and hybrid settings com pared to in-person arrangements. This is aligned with emerging literature suggesting that employees increasingly prefer flexible work arrangements and may feel more psychologically safe in these contexts. Notably, and contrary to our expectations, psychological safety was significantly higher when organizations made the decision to implement remote or hybrid arrangements rather than when employees chose the- se arrangements independently. This counterintuitive finding suggests that organizational commitment to teleworking may demonstrate trust and support to employees, potentially activating reciprocity mechanisms within the psychological contract.

Several theoretical explanations emerged from our results. First, organizational decisions to implement remote work may be interpreted by employees as a demonstration of institutional trust, which employees reciprocate through enhanced feelings of safety and belonging. Second, when organizations uniformly adopt remote or hybrid policies, employees may experience less fear of missing out (FoMO) compared to situations where individual choices create disparate work arrangements within teams. Third, organizational implementation of flexible arrangements may require and foster the open communication channels necessary for psychological safety to flourish. Conversely, organizational decisions to maintain exclusively in-person arrangements may be perceived negatively in the current context, potentially signaling mistrust or a lack of organizational progressiveness.

In this sense, this research provides valuable insights to the relationship between work arrangements and psychological safety in contemporary organizations and it offers important theoretical contributions by examining psychological safety in the context of telework. In a practical perspective, our findings suggest that organizations that genuinely committed to structured remote or hybrid work policies may cultivate higher levels of psychological safety compared to those that leave arrangement decisions solely to individual employees. This has significant implications for organizations navigating post-pandemic work arrangements and seeking to maintain psychologically safe work environments.

In conclusion, this study emphasizes the need for structured, organization-wide approaches to support psychological safety in modern work contexts. Organizations that proactively and uniformly implement teleworking arrangements, accompanied by appropriate communication and support may be better positioned to foster psychologically safe environments. As the world of work continues to evolve, understanding how organizational policies and employee autonomy interact to shape psychological safety will remain crucial for both scholarly inquiry and organizational practice.

## References

[B1] Deci E. L., Olafsen A. H., Ryan R. M (2017). Self-Determination Theory in Work Organizations: The State of a Science. Annual Review of Organizational Psychology and Organizational Behavior.

[B2] Deci E. L., Ryan R. M (1985). Intrinsic motivation and self-determination in human behavior. Contemporary Sociology.

[B3] Dzandu M., Theophilus I., Issa D (2023). Exploring the relationship between personal and work characteristics of project managers and psychological safety in virtual teams. Procedia Computer Science.

[B4] Edmondson A. C (1999). Psychological Safety and Learning Behavior in Work Teams. Administration Science Quarterly.

[B5] Edmondson A.C, West M.A., Tjosvold D., Smith K.G. (2003). International Handbook of Organizational Teamwork and Cooperative Working.

[B6] Edmondson A. C, Kramer R.M., Cook K.S. (2004). Trust and distrust in organizations: Dilemmas and approaches.

[B7] Edmondson A. C., Lei Z (2014). Psychological safety: The history, renaissance, and future of an interpersonal construct. Annual Review of Organizational Psychology and Organizational Behavior.

[B8] Edmondson A. C (2019). The fearless organization: Creating psychological safety in the workplace for learning, innovation, and growth.

[B9] Edmondson A. C., Bransby D. P (2023). Psychological Safety Comes of Age: Observed Themes in an Established Literature. Annual Review of Organizational Psychology and Organizational Behavior.

[B10] Gari F., Dimas I., Lourengo P. R., Rebelo T, Silva P. da, Jorge S., Sá P. (2020). Emerging Topics in Management Studies.

[B11] ILO (2020). United Nations Digital Library System.

[B12] Kibaroglu G.G., Ululan A.R., Basim H. N (2025). How does Job Passion Stimulate Job Crafting Behaviours? The Role of Psychological Safety. J. East Eur. Manag. Stud.

[B13] Kossek E. E., Thompson R. J., Lautsch B. A (2015). Balanced Workplace Flexibility: Avoiding the Traps. California Management Review.

[B14] Lechner A., Tobias Mortlock J (2021). How to create psychological safety in virtual teams. Organizational Dynamics.

[B15] Newman A., Donohue R., Eva N (2017). Psychological safety: A systematic review of the literature. Human Resource Management Review.

[B16] Peters P., Ligthart P. E. M., Bardoel A., Poutsma E (2016). “Fit” for telework'? Cross-cultural variance and task-control explanations in organizations' formal telework practices. The International Journal of Human Resource Management.

[B17] Przybylski A. K., Murayama K., DeHaan C. R., Gladwell V (2013). Motivational, emotional, and Behavioral Correlates of Fear of Missing out. Computers in Human Behavior.

[B18] Reisinger H., Fetterer D (2021). Forget Flexibility. Your Employees Want Autonomy. Harvard Business Review.

[B19] Rousseau D. M., Sitkin S. B., Burt R., Camerer C (1998). Not so different after all: A cross-disciplinary view of trust. Academy of Management Review.

[B20] Rousseau D. M (1989). Psychological and implied contracts in organizations. Employee Responsibilities and Rights Journal.

[B21] Ryan R. M., Deci E. L (2017). Self-determination theory: Basic psychological needs in motivation, development, and wellness.

[B22] Ryan R (2009). Self-determination theory and well-being. Social Psychology.

[B23] Ryan R.M., Deci E.L (2000). Self-determination theory and the facilitation of intrinsic motivation, social development, and well-being. American Psychologist.

[B24] Sjoblom K., Juutinen S., Makikangas A (2022). The Importance of Self-Leadership Strategies and Psychological Safety for Well-Being in the Context of Enforced Remote Work. Challenges.

[B25] Smith B (2022). Psychological Safety at Work: The Remote Kids are Alright (Maybe Even Better). MeQuilibrium.

[B26] Stegmann S., Van Dick R., Ullrich J., Charalambus J., Menzel B., Egold N (2010). Der Work Design Questionnaire: Vor-stellung und erste Validierung einer deutschen Version. Z. Arb.-Und Organ. AO.

[B27] Tanzi A (2022). Remote Work Is a Choice, Not a Necessity, for Most, Pew Poll Shows. Bloomberg.com.

[B28] Theurer C. P., Tumasjan A., Welpe I. M (2018). Contextual work design and employee innovative work behavior: when does autonomy matter?. Plos One.

[B29] Tusl M., Brauchli R., Kerksieck P., Bauer G. F (2021). Impact of the COVID-19 crisis on work and private life, mental well-being and self-rated health in German and Swiss employees: a cross-sectional online survey. BMC Public Health.

